# Photoreceptor Differentiation following Transplantation of Allogeneic Retinal Progenitor Cells to the Dystrophic Rhodopsin Pro347Leu Transgenic Pig

**DOI:** 10.1155/2012/939801

**Published:** 2012-04-09

**Authors:** H. Klassen, J. F. Kiilgaard, K. Warfvinge, M. S. Samuel, R. S. Prather, F. Wong, R. M. Petters, M. la Cour, M. J. Young

**Affiliations:** ^1^Gavin Herbert Eye Institute, Ophthalmology Research, University of California-Irvine, Irvine, CA 92697-4390, USA; ^2^Department of Ophthalmology, Glostrup Hospital, SUND, University of Copenhagen, 2600 Copenhagen, Denmark; ^3^Warfvinge Science Support and Department of Ophthalmology, Lund University, SE, 221 84 Lund, Sweden; ^4^Division of Animal Sciences, University of Missouri-Columbia, Columbia, MO 65211, USA; ^5^Duke Eye Center, Duke University, Durham, NC 27710, USA; ^6^Department of Animal Science, North Carolina State University, Raleigh, NC 27695, USA; ^7^Minda de Gunzburg Center for Ocular Regeneration, Schepens Eye Research Institute, Boston, MA 02114, USA

## Abstract

*Purpose*. Transplantation of stem, progenitor, or precursor cells has resulted in photoreceptor replacement and evidence of functional efficacy in rodent models of retinal degeneration. Ongoing work has been directed toward the replication of these results in a large animal model, namely, the pig. *Methods*. Retinal progenitor cells were derived from the neural retina of GFP-transgenic pigs and transplanted to the subretinal space of rhodopsin Pro347Leu-transgenic allorecipients, in the early stage of the degeneration and the absence of immune suppression. *Results*. Results confirm the survival of allogeneic porcine RPCs without immune suppression in the setting of photoreceptor dystrophy. The expression of multiple photoreceptor markers by grafted cells included the rod outer segment-specific marker ROM-1. Further evidence of photoreceptor differentiation included the presence of numerous photoreceptor rosettes within GFP-positive grafts, indicative of the development of cellular polarity and self-assembly into rudiments of outer retinal tissue. *Conclusion*. Together, these data support the tolerance of RPCs as allografts and demonstrate the high level of rod photoreceptor development that can be obtained from cultured RPCs following transplantation. Strategies for further progress in this area, together with possible functional implications, are discussed.

## 1. Introduction

As a group, degenerative diseases of the retina constitute a significant source of visual disability, particularly in the developed world, and yet current therapeutic options are quite limited. For instance, the loss of photoreceptor cells, as seen in the later stages of retinitis pigmentosa and macular degeneration, results in permanent visual deficits for which no restorative treatment is available. Nevertheless, the notion that photoreceptor cells might be replaceable in the therapeutic setting has been given recent support by experimental work in animal models.

Work in the rat first showed that transplanted neural progenitor cells could migrate into the host retina, take up residence within the cellular laminae of this tissue, and exhibit morphological signs of integration into the local cytoarchitecture [1–3]. Subsequent studies reported that similar cells could be derived from the developing neural retina of both rats [4] and mice [5, 6] and that these retinal progenitor cells (RPCs) could express photoreceptor markers, including recoverin and rhodopsin, and rescue light sensitivity following engraftment in the host retina.

More recent work has shown that many of the results obtained using progenitor cell transplantation in rodents also apply to other mammalian species, including the Brazilian opossum [7], the pig [8–10], and the cat [9, 10]. In some of this work, particularly following the use of brain-derived progenitors as donor cells, the evidence for retinal integration was substantial and yet the evidence of photoreceptor differentiation was limited [7, 9–13].

Prior work in the pig has shown profuse expression of photoreceptor markers by grafted retinal progenitor cells, yet the full extent of donor cell differentiation was difficult to determine, in part due to limited visualization of donor cell morphology [9, 10]. In addition, previous work in the pig made use of retinal injury models, whereas a model of photoreceptor degeneration has also been generated in the pig [14–16]. Here we investigate the fate of RPCs derived from GFP-transgenic pigs following transplantation to the subretinal space of transgenic rhodopsin Pro347Leu allorecipients.

## 2. Methods

### 2.1. Donor Animals and Cells

Two timed-pregnant NT5 GFP-transgenic sows [17] were sacrificed at 45 days gestation, on separate occasions, to provide fetal tissue as the starting point for the derivation of GFP-transgenic porcine retinal progenitor cells (gpRPCs). Details of the tissue harvest and cell derivation procedures were otherwise similar to those previously described [9, 10]. Briefly, the immature neural retina was dissected free from each fetal eye (excluding the optic nerve head and ciliary margin), the retinal tissue was pooled and subjected to repeated cycles of enzymatic digestion followed by seeding into tissue culture flasks in neurobasal media supplemented with B-27 and 20 ng/mL EGF and bFGF. FBS (10%) was included overnight, but then removed with all subsequent feedings being serum-free. These cells of retinal origin were then expanded under serum-free culture conditions thereby selectively enriching for proliferating progenitors.

The resulting cultures were defined as RPCs and these cells have been further characterized, as previously reported [9, 10, 18]. Like brain-derived porcine neural progenitors [19], these cells are nestin-, sox2-, and Ki-67-positive, and small subpopulations express neuronal markers and the glial marker GFAP. Unlike brain-derived neural progenitors, proliferating RPC cultures contain subpopulations of recoverin-positive profiles [5, 6], whereas rhodopsin expression is rare and ROM-1 undetectable. Differentiating RPCs give rise to rod photoreceptor cells but not oligodendrocytes.

Cells were passaged for approximately 4–6 weeks prior to transplantation. The cells were grown as monolayers and dissociated with each passage, but prior to transplantation were allowed to form nascent spherical aggregates, and the smaller aggregates were preferentially collected for use as donor material. The reason for adopting this last approach is that fully dissociated RPCs exhibit poor survival rates following transplantation, whereas large spherical aggregates can be associated with glial differentiation within the sphere core [19].

### 2.2. Recipient Animals

Recipient animals used in the present experiments were 15 transgenic rhodopsin Pro347Leu transgenic swine (from 2 litters: age 6 and 9 weeks) with a known retinal dystrophic phenotype [15] as well as 1 nontransgenic littermate (age: 6 weeks), which served as a normal control.

### 2.3. Transplant Surgery

The transplantation technique was similar to that previously described in pigs [8–10, 18]. Briefly, recipient animals underwent preanesthesia, endotracheal intubation, and general anesthesia. The left pupil was dilated. A standard three port pars plana vitrectomy was performed. The posterior hyaloids were detached and the central vitreous removed in all cases. A retinal bleb was elevated in the area centralis by the subretinal injection of 0.25–0.5 mL BSS through a 41-gauge cannula (ref. 1270; DORC International BV, Zuidland, the Netherlands). A small retinotomy was made by gentle endodiathermy of the detached retina. GFP+ cells (approximately 5–10 × 10^6^ cells) were injected either as spheres or as single cell suspension through the retinotomy and into the retinal bleb using a 27-gauge silicon-tipped needle. Immediate reflux of some cells into the vitreous cavity was observed in some animals. A small air bubble was placed in the subretinal bleb under the retinotomy to prevent further reflux of cells after withdrawal of the needle. Sclerotomies and conjunctiva were sutured with 7–0 vicryl. Lateral canthal incisions were sutured with 6–0 vicryl. The pigs were examined by ophthalmoscopy 1-2 days after surgery.

The research protocol used was previously reviewed and approved by the Danish Animal Experiment Inspectorate, the North Carolina State University IACUC, and completed in accordance with the ARVO statement for the Use of Animals in Ophthalmic and Vision Research.

### 2.4. Tissue Processing

Eyes were enucleated following euthanasia by overdose of intravenous pentobarbital at the termination of the experiment. The survival time for the first litter (6 dystrophic animals, 1 normal control) was 5 weeks posttransplantation (age 11 weeks), while that of the second litter (9 animals) was 9 weeks (age 18 weeks). Globes were placed in 4% paraformaldehyde (PFA) for 10–20 minutes. The anterior segment and the lens were then removed and the posterior segment was postfixed for 2 hours in 4% PFA, with subsequent rinsing in rising concentrations of sucrose containing Sörensen's phosphate buffer. A horizontal cut was made which extended from the temporal retinal margin to 2-3 mm nasal to the optic disc, thus comprising the temporal ciliary margin, the area centralis, and the optic disc. The tissues were embedded in a gelatin medium and serially sectioned at 12 *μ*m on a cryostat. During the sectioning process, every fifteenth section was examined by epifluorescence microscopy for GFP+ cells, and every tenth slide was stained with Hematoxylin-Eosin (Htx-Eosin).

### 2.5. Immunohistochemistry

The retinal sections were exposed to primary anti-sera (see Table 1) in a moist chamber for 16–18 hours, 4°C, followed by rinsing in 0.1 M phosphate-buffered saline (PBS) with 0.25% Triton-X-100. Sections were then incubated with secondary Alexa 409 (recoverin; 1 : 400, Invitrogen, La Jolla, CA) or Texas Red-conjugated antibodies (ROM-1 and rhodopsin, 1 : 200, Jackson Immunoresearch, West Grove, PA) for 1-2 hrs at room temperature in the dark. Normal eyes, processed in parallel, were used as controls. In addition, negative controls with omission of the primary anti-sera were performed. The specimens were examined with an epifluorescence microscope. Colocalization of Texas Red-labeled primary antibodies and GFP+ cells was assessed by superimposition of separate digital images of each fluorochrome.

Sections were frequently stained with chicken anti-GFP (with FITC secondary antibody, 1 : 200, Jackson Immunoresearch, West Grove, PA), either in order to examine the quality of the GFP expression or to reveal possible downregulated grafted cells. The endogenous GFP expression was always high and enhancing with anti-GFP resulted in blurring of the cell boundaries. The GFP staining did not appear to reveal more surviving cells.

## 3. Results

**Figure 1:**
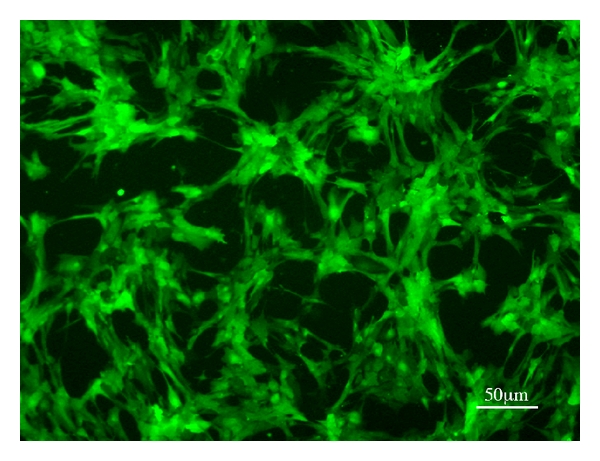
Donor porcine retinal progenitor cells (RPCs). RPCs isolated from GFP-transgenic donors at 45d gestational age and expanded in culture in the presence of EGF and bFGF. The cells grew as a monolayer and strongly expressed the GFP reporter gene. No tendency toward rosette formation is seen.

Progenitor cells from the fetal porcine retina proliferated rapidly in culture, as previously described [9, 10, 18]. Cultured GFP-transgenic pRPCs maintained extensive reporter gene-associated fluorescence and did not show evidence of rosette formation under proliferation conditions (Figure 1).

**Figure 2:**
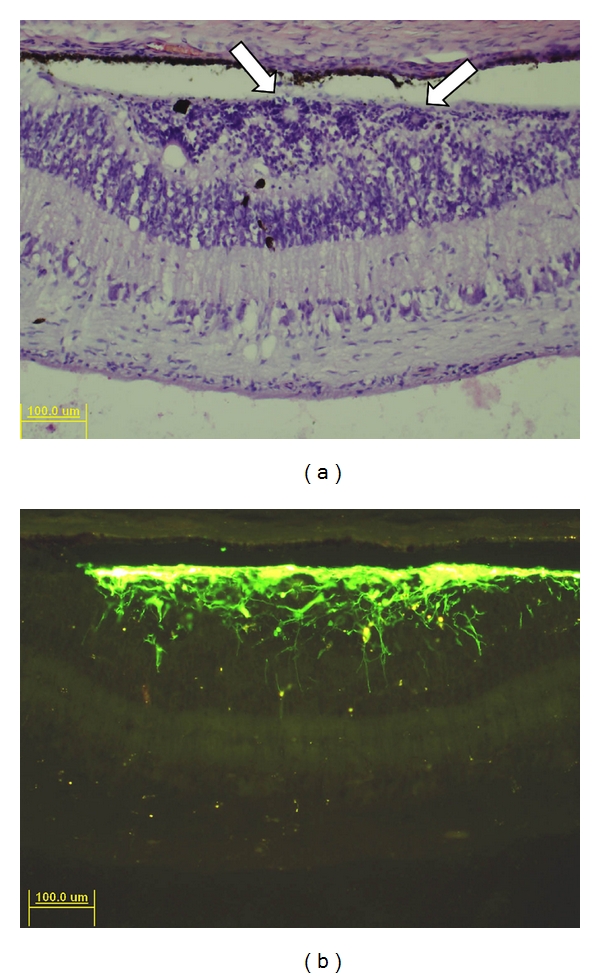
Engraftment of RPCs in rhodopsin Pro347Leu recipient. Recipient was 6 weeks of age at time of transplantation. (a) H&E stained section through the graft site 5 weeks after transplantation shows a cellular mass in the subretinal space of presumptive donor origin. Artifactual detachment of the retina reveals the cellular mass to be adherent to the outer surface of the retina, in preference to the adjacent RPE layer. A number of photoreceptor rosettes are present within the cellular mass (arrows), as well as some focal pigment profiles, some of which are also found within the adjacent host neural retina. (b) Fluorescence imaging shows intense GFP-associated immunoreactivity in the subretinal space, corresponding to the cellular mass noted with H&E, above, thereby confirming the identity and location of the graft. A presumptive rosette is visible within the center of the graft, in a location similar to that seen in the H&E section. The majority of GFP+ cells remain in the subretinal mass; however, a number of donor cells are visible within the host retina and these have processes that exhibit a high degree of radial orientation.

Following transplantation to nonimmunosuppressed juvenile rhodopsin Pro347Leu transgenic pigs, GFP+ donor cells could be readily identified at the site of transplantation (Figure 2). Strong nuclear staining and classical rosettes were apparent within the cellular aggregate of the graft. There were also a few pigmented profiles present within the subretinal graft and host outer nuclear layer (ONL; Figure 2(a)). Despite a few limited indications of prior surgical intervention, there was no clinical or histological evidence of inflammatory or immune responses in recipient eyes.

**Figure 3:**
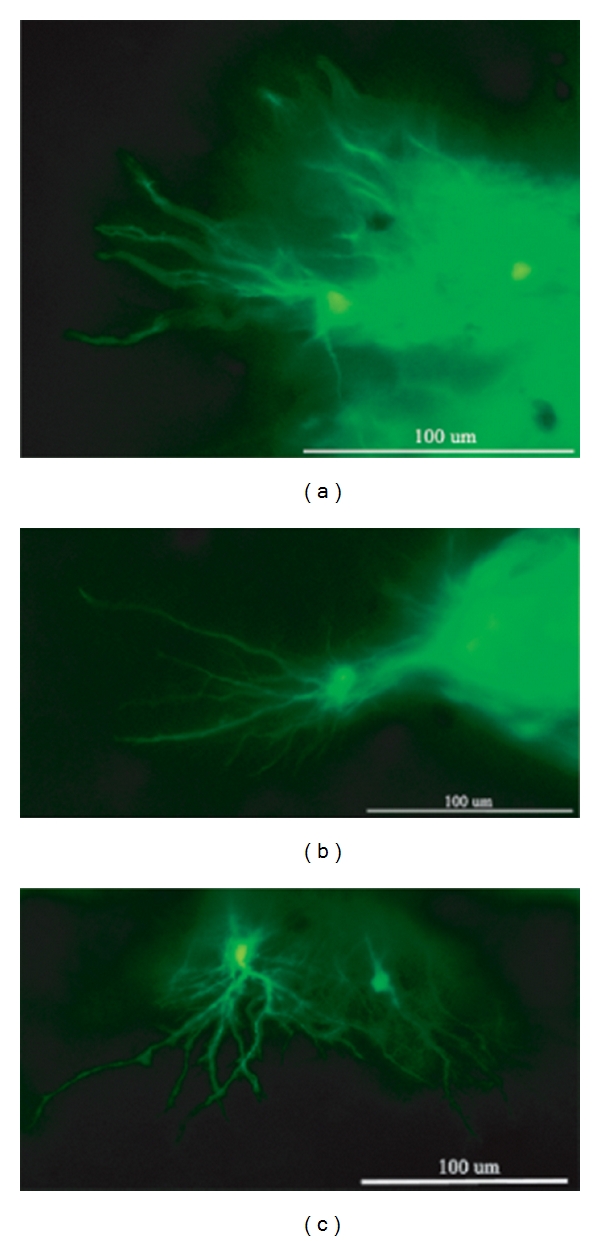
Arborization of grafted cells in dystrophic recipient retina. Recipient was 9 weeks of age at time of transplantation and experiment terminated 9 weeks later. Photomicrograph of a retinal wholemount viewed *en face* using fluorescence microscopy. Image reveals GFP+ profiles within the retina of rhodopsin Pro347Leu recipient. Inspection of regions near the edge of the graft allows visualization of individual donor-derived cells and reveals extension of elaborate processes in the plane of focus.

**Figure 4:**
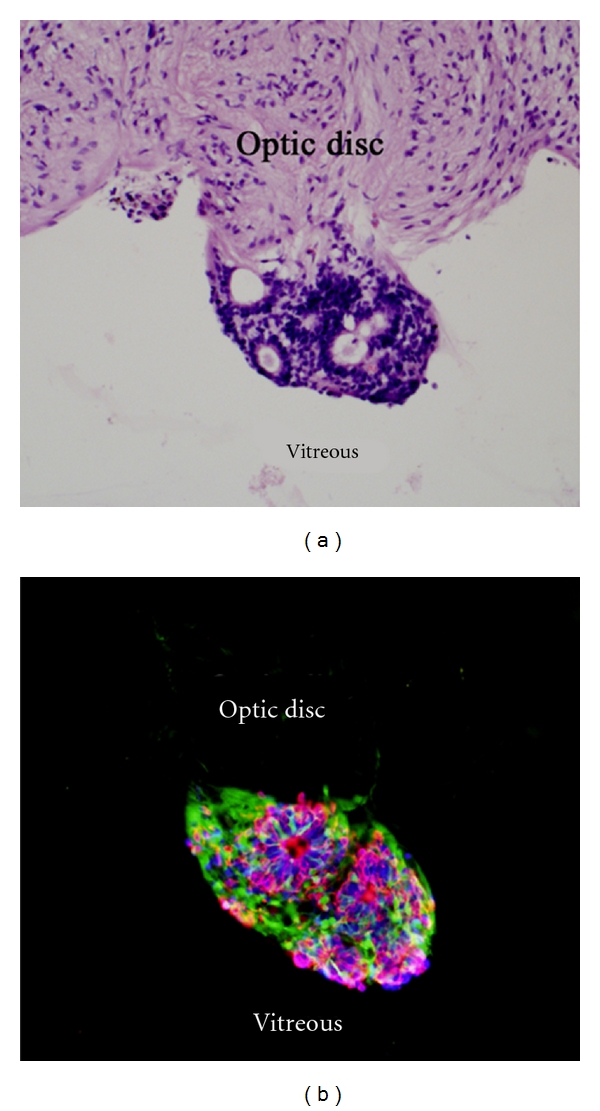
Grafted cells form rosettes and express photoreceptor markers. Recipient was 6 weeks of age at time of transplantation and experiment terminated 5 weeks later. The H&E section (a) shows a cluster of grafted cells adhering to vitreal surface of the host optic nerve head, likely a result of reflux around the time of surgery. This cluster contains multiple rosettes of the type formed by photoreceptor cells, with darkly staining nuclei densely packed in a peripheral rim and lighter staining material in the rosette's core. Fluorescence imaging (b) confirms the cluster to be GFP+ and therefore of donor origin. Immunolabeling for rhodopsin (red) and recoverin (blue) demonstrates photoreceptor marker expression by a subset of grafted cells. Cells expressing photoreceptor markers are predominantly found within rosettes.

Donor cells were predominantly located in the subretinal space directly adjacent to the RPE layer, while a subset of GFP+ profiles migrated or extended processes into the host ONL. Cells in the subretinal space frequently extended processes perpendicular to the retina or with random orientations, whereas GFP+ processes within the host ONL exhibited predominantly radial orientations (Figure 2(b)). Donor profiles within the neural retina at a distance from the graft site showed extensive horizontal branching, as visualized in retinal wholemounts (Figure 3). In one case, a cluster of donor cells was identified on the vitreal surface of the optic nerve head, presumably as a result of reflux through the retinotomy at the time of subretinal injection (Figure 4). These cells had formed a rudiment of retinal tissue that appeared to be fused to the optic nerve tissue, as seen in work with embryonic retinal tissue grafts [20].

There was evidence of widespread differentiation of donor RPCs along the photoreceptor lineage following transplantation to the eye of dystrophic allorecipients. This data included both morphological considerations and marker expression. In H&E stained sections, cells within the graft predominantly exhibited small, densely packed nuclei which closely resembled adjacent photoreceptors of the host ONL (Figure 2(a)). In addition, there were numerous rosettes consisting of a dense ring of nuclei surrounding a pale central core. These were of the type known to be formed by photoreceptor cells under a variety of circumstances following disruption and spontaneous reorganization of the ONL [20]. In addition, the cells could be identified as being of donor origin based on graft location (subretinal space, vitreous cavity) and comparison to GFP expression patterns (Figures 2(b) and 4).

**Figure 5:**
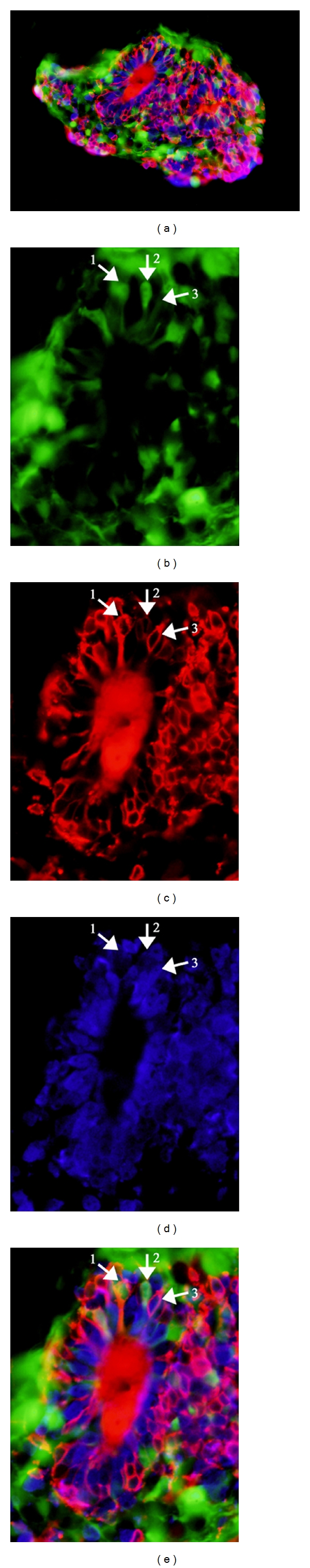
Photoreceptor marker expression within a rosette of donor origin. Recipient was 6 weeks of age at time of transplantation and experiment terminated 5 weeks later. (a) Immunohistochemical labeling of the same RPC graft was shown in previous figure. Endogenous GFP fluorescence (green) was present in many cells of the graft, but less so within the rosettes. The largest, most well formed of the rosettes was selected for more detailed evaluation of individual marker expression patterns. (b) GFP labeling was most notable around the periphery of the rosette; however, GFP+ profiles were also integrated into this structure, with small peripheral soma and straight process oriented towards the core of the rosette (arrow 1). An adjacent region (arrow 2) shows a similar cell with a less clearly defined process. A third cell (arrow 3), does not express GFP. (c) Rhodopsin labeling (red) is strongest in the central core of the rosette. In addition, multiple profiles exhibiting rod photoreceptor morphology can be seen to contribute to the rim of the rosette, and these have cell bodies located around the periphery and processes directed inward where they merge with the central core. The arrows point to the same profiles as before, in this case showing strong rhodopsin labeling (arrow 1), no rhodopsin (arrow 2), and moderate rhodopsin (arrow 3). (d) Recoverin (blue) labeling was dense in areas populated by rhodopsin-positive cells and revealed a dense ring of recoverin-positive cells central to the rhodopsin-positive somata, although this labeling did not extend into the core of the rosette despite some marginal overlap at the interface. (e) The merged image reveals that much of the rhodopsin colabels recoverin-positive cells, whereas many recoverin-positive cells do not appear to colabel with rhodopsin. The core is exclusively positive for rhodopsin, as previously noted.

**Figure 6:**
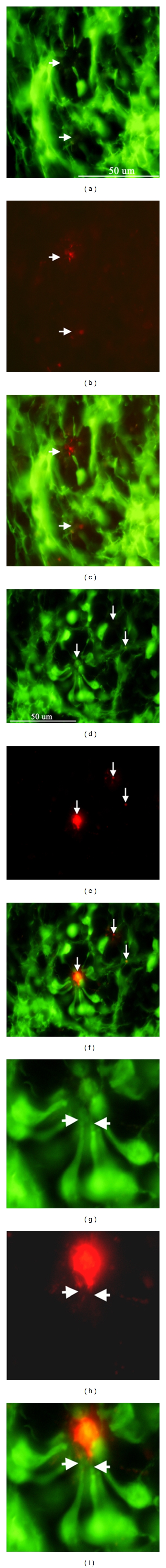
Expression of ROM-1 within rosettes of the graft. Recipient was 6 weeks of age at time of transplantation and experiment terminated 5 weeks later. Immunohistochemical labeling for rod outer segment marker-1. Panels in the left column of Figures (a, d, g) show endogenous GFP transgene expression (green) of donor cells, panels in the central column (b, e, h) show ROM-1 expression (red), and panels in the right column (c, f, i) show the merged images comprised of the 2 preceding panels of the same row. (a) The widespread GFP expression within the graft is periodically interrupted by rounded lacunae (arrows), where (b) ROM-1 labeling can be found. Merged image (c) shows that regions of ROM-1 labeling are encircled by inwardly directed straight processes, thereby indicating that ROM-1 corresponds to the core region of the rosettes, which is the predicted location for rod outer segments in this type of structure. These findings are corroborated by examination of another rosette (d) in which there is a greater number of GFP+ cells with photoreceptor morphology, the processes of which are directed inward toward a small central core. (e) ROM-1 labeling (arrows) predominantly localizes to the core region of the rosette. (g–i) Examination of the same rosette at higher power allows visualization of the limit of GFP expression (arrows) within some of the processes of donor-derived photoreceptor-like cells, in juxtaposition to the adjacent localization of ROM-1 labeling.

Further support for photoreceptor differentiation was obtained by analysis of marker expression. Grafted cells showed immunohistochemical evidence for expression of multiple photoreceptor-associated markers. Cells of the grafts, including cells within rosettes, showed expression of recoverin and rhodopsin, with a notable degree of double-labeling for these markers (Figures 4 and 5). These markers were expressed by donor cells within grafts, regardless of location within either the subretinal space or vitreal surface of the optic nerve. The rod photoreceptor outer segment-specific marker ROM-1 was also expressed by rosette-forming grafted cells (Figure 6).

In terms of marker expression patterns, GFP was evident in many grafted cells but was somewhat less expressed within rosettes and was excluded from the central core of these structures (Figures 4 and 5), as might be expected based on differential protein trafficking. Conversely, rhodopsin expression was more predominant within rosettes and was notable for being highly concentrated in the central core. Recoverin was also more highly expressed within the rosettes, albeit with a different pattern than rhodopsin. Recoverin appeared to be excluded from all but the periphery of the rosette central core (Figure 5). ROM-1, a marker specifically associated with rod outer segment disc membranes, was exclusively expressed in the rosette central core (Figure 6).

## 4. Discussion

A major challenge faced during the development of therapeutic applications for stem cells is the requirement for reliable tissue-specific differentiation of grafted cells. This is particularly true in the setting of the retina, owing both to the need for molecular transduction of photic stimuli as well as the requirement for a highly organized and optically transparent tissue. Here we show that cultured RPCs are capable of attaining a high level of photoreceptor development following transplantation to the retina of a large mammal. In this model, grafted RPCs differentiated into photoreceptor-like profiles in sufficient quantities and with sufficient structural polarity and cytological affinity to self-organize into rosettes. Marker expression closely followed normal patterns, supporting the conclusion that these donor cells had differentiated into morphological photoreceptors.

Previous work with stem [21], progenitor [5, 6], and precursor [22] cell transplantation has shown that various degrees of morphological photoreceptor development can be achieved by way of this approach in rodents. Moreover, these same studies have provided evidence of functional improvements in host vision, relative to controls. Although rodent recipients are frequently used, these types of cells have been derived from human sources [5, 6, 21, 23–26]. The question, therefore, arises as to whether allogeneic RPC transplantation represents a viable method for treatment of human retinal conditions. One way to approach this question prior to human trials is the use of large animal models for replication of the rodent data under conditions more equivalent to those faced clinically. One such model that is gaining in popularity is the pig. In terms of retinal degeneration, the pig model is enhanced by the availability of swine transgenic for the reporter gene GFP for use as donors [17, 18] and retinal degenerative swine for use as recipients [14–16]. We have previously shown that progenitor cells can be isolated from the porcine brain [19] and retina [9, 10], including from GFP-transgenic donors [18], and that porcine RPCs are capable of expressing photoreceptor markers, both in vitro and after transplantation to the subretinal space [9, 10]. Here we provide additional evidence that grafted RPCs undergo a high level of photoreceptor differentiation after transplantation to the dystrophic pig eye.

We found that porcine RPCs expressed photoreceptor markers after transplantation to the subretinal space, yet photoreceptor differentiation was also observed in a grafted cluster of cells that adhered ectopically to the optic nerve head. The unintended location of this cluster is likely a consequence of the misdirection of grafted neurospheres, at or near the time of surgery, presumably due to reflux from the subretinal space through the retinotomy, as is known to occur. In this instance, the potential for confusion with host cells was eliminated by endogenous GFP reporter gene expression. Interestingly, this data supports the concept that RPCs are capable of spontaneously differentiating into photoreceptors at relatively high yield without the need for microenvironmental cues such as might be obtained from engraftment in the host retina. In addition, the photoreceptor cells generated within the graft exhibited sufficient polarity to self-organize into rosettes. This particular cluster contained multiple rosettes of the type typical of those formed by photoreceptor cells after various perturbations of the retina, wherein the nuclei are densely packed around the periphery and the inner and outer segments are oriented inward to form the rosette's core. These types of highly organized rosettes can be viewed as focal tissue rudiments, each attempting to recapitulate the structure of the outer nuclear layer (ONL), albeit in a manner lacking global continuity. Rosettes of this type are to be distinguished from the more primitive Flexner Wintersteiner rosettes seen in a number of neoplastic conditions.

The functional capabilities of the grafted cells were not assessed; however, prior work with embryonic retinal transplants in rodents has demonstrated that rosettes of similar morphology are capable of detecting light and providing limited light-mediated behaviors, even in the absence of an RPE layer [20, 27–29]. That said, the abnormal topography of a rosette obviously limits the potential for spatial vision. The point to be made is that although rosette formation poses a limit to spatial vision, it does not rule out the potential for functionality at the level of the graft-derived photoreceptors. Photoreceptors in the cluster attached to the optic nerve would not be expected to integrate with the host visual pathways; however, cells transplanted to the subretinal space may have done so although this possibility was not demonstrated in the current study. It could be that such integration did not occur, or it may have occurred but been obscured by the tendency of donor cells to downregulate GFP expression as they differentiated into rhodopsin- and recoverin-expressing photoreceptors. This latter tendency is attributable to the CMV promoter used to drive GFP expression in the transgenic donor pigs. Use of an alternate promoter could provide a solution to this problem [30].

Appropriately localized expression of the rod-specific marker ROM-1 seen in the current study provides additional evidence of the high level of photoreceptor differentiation obtainable from cultured RPCs. The presence of ROM-1 in the core of rosettes is consistent with the development of rod outer segments (ROS) by the donor-derived photoreceptors. The survival of the grafted cells seen in the current study, in the absence of immune suppression, provides additional confirmation for the high level of tolerance shown to allogeneic RPCs following introduction to both the vitreous cavity and subretinal space of a retinal dystrophic large mammal. Less certain is the contribution of the transgenic rhodopsin Pro347Leu background to the integration of donor cells. RPCs, like many other types of neural progenitor and precursor cells, are known to display an evident tropism for areas of degeneration, trauma, or disease. It was anticipated that the use of dystrophic hosts would result in enhanced intraretinal integration by grafted RPCs. GFP+ profiles did exhibit radially oriented integration into the host ONL and extension of elaborater processes in the orthogonal plane; however, the identity of the integrated cells remains to be determined, as does the question of graft-host connectivity.

The pig has emerged a preferred species for modeling of surgical procedures, including ocular applications. The increasing availability of transgenic swine and recent sequencing of the porcine genome provide additional advantages to the use of this model. The current study illustrates the value of the porcine model in the translational development of regenerative strategies and, in particular, intraocular stem cell transplantation.

## Figures and Tables

**Table 1:** Primary antibodies used for immunohistochemistry.

Name	Host	Dilution	Detects	Supplier
Recoverin	Rabbit	1 : 10.000	Rods, cones, some bipolar cells	Kind gift of Dr. A Dizhoor, Detroit, MI
Rho4D2	Mouse	1 : 100	Rods	Kind gift of Dr. RS Molday, Vancouver, Canada
ROM-1	Mouse	1 : 10	Rod outer segments	Kind gift of Dr. RS Molday, Vancouver, Canada
GFP	Chicken	1 : 5000	Green fluorescent protein	Chemicon, Temecula, CA
